# Urinary adiponectin and albuminuria in non-diabetic hypertensive patients: an analysis of the ESPECIAL trial

**DOI:** 10.1186/s12882-015-0124-3

**Published:** 2015-08-01

**Authors:** Seung Seok Han, Eunjin Bae, Shin Young Ahn, Sejoong Kim, Jung Hwan Park, Sung Joon Shin, Sang Ho Lee, Bum Soon Choi, Ho Jun Chin, Chun Soo Lim, Suhnggwon Kim, Dong Ki Kim

**Affiliations:** Department of Internal Medicine, Seoul National University College of Medicine, 101 Daehakro, Jongno-gu, Seoul 110-744 South Korea; Department of Internal Medicine, Seoul National University Bundang Hospital, Gyeonggi-do, 463-707 South Korea; Department of Internal Medicine, Konkuk University School of Medicine, Seoul, 143-729 South Korea; Department of Internal Medicine, Dongguk University Ilsan Hospital, Gyeonggi-do, 410-773 South Korea; Department of Internal Medicine, Kyung Hee University Medical Center, Seoul, 134-727 South Korea; Department of Internal Medicine, Seoul St. Mary’s Hospital, Seoul, 137-701 South Korea; Department of Internal Medicine, Seoul National University Boramae Medical Center, Seoul, 156-707 South Korea

**Keywords:** Adiponectin, Albuminuria, Biomarker, Kidney, Urine

## Abstract

**Background:**

Although adiponectin levels have been reported to be correlated with albuminuria, this issue remains unresolved in non-diabetic hypertensive subjects, particularly when urinary adiponectin is considered.

**Methods:**

Urinary adiponectin levels were examined using an enzyme-linked immunosorbent assay in 229 participants. who used olmesartan as a hypertensive agent. Their albuminuria levels were measured for 16 weeks after randomization and initiation of conventional or intensive diet education. Linear or logistic regression models were applied, as appropriate, to explore the relationship with albuminuria itself or its response after the intervention.

**Results:**

Urinary adiponectin levels were positively related to baseline albuminuria level (*r* = 0.529). After adjusting for several covariates, the adiponectin level was associated with the albuminuria level (*β* = 0.446). Among the 159 subjects with baseline macroalbuminuria, the risk of consistent macroalbuminuria (> 300 mg/day) at 16 weeks was higher in the 3^rd^ tertile of adiponectin than in the 1^st^ tertile (odds ratio = 6.9), despite diet education. In contrast, among all subjects, the frequency of the normoalbuminuria achievement (< 30 mg/day) at 16 weeks was higher in the 1^st^ tertile than in the 3^rd^ tertile (odds ratio = 13.0).

**Conclusions:**

Urinary adiponectin may be a useful biomarker for albuminuria or its response after treatment in non-diabetic hypertensive patients.

**Electronic supplementary material:**

The online version of this article (doi:10.1186/s12882-015-0124-3) contains supplementary material, which is available to authorized users.

## Background

Adiponectin, an adipokine, has several benefits related to its insulin-sensitizing, anti-inflammatory, and anti-atherosclerotic effects [1]. This peptide has primarily been studied in certain disease subsets, such as metabolic syndrome, obesity, and diabetes mellitus [2], and this trend may have occurred because adiponectin is secreted by adipocytes. Recently, the role of adiponectin in other types of kidney disease (e.g., non-diabetic kidney disease) was discussed [3, 4]. The correlation between adiponectin and non-diabetic kidney disease is plausible because the pathophysiology in most kidney diseases is based on inflammation [5], and some kidney diseases are strongly attributable to the spectrum of obesity-related diseases [6]. Additionally, the direct effect of adiponectin on the kidney, as observed in experimental studies, supports this relationship [7].

Previous clinical studies have revealed that the blood concentration of adiponectin is related to kidney injury, including albuminuria. With the beneficial effect of adiponectin in mind, low blood adiponectin potentially serves as a biomarker for a high risk of albuminuria [8]. However, the opposite relationship between them has been documented in certain types of disease, such as type 1 diabetes [9]. Additional paradoxical findings where the blood adiponectin level is higher in patients with chronic kidney disease or end-stage renal disease than in counterpart subjects further complicate our understanding [10]. Although the adiponectin feedback mechanism or dependent relationship with other mediators may be responsible for the inconsistent results, it is not easy to measure blood adiponectin as a biomarker across several disease subsets.

In contrast to blood adiponectin, a positive correlation between urinary adiponectin and albuminuria has been consistently demonstrated in previous studies. However, it is too early to determine whether urinary adiponectin is a consistent biomarker for the risk of albuminuria because most studies included diabetic patients [11], and the counterpart studies including non-diabetic patients are scarce, had low sample sizes, or were restricted to only one disease subtype [3, 12, 13]. Here, we first addressed the relationship between urinary adiponectin and albuminuria in a prospective cohort of non-diabetic hypertensive patients. Then, the issue of whether an individual’s baseline level of urinary adiponectin can predict clinically implicated albuminuria after 16 weeks was addressed.

## Methods

### Participants and data collection

The study protocol complies with the Declaration of Helsinki and received full approval from the institutional review board at Seoul National University Hospital (no. H-1111-089-387). The present study was prospectively conducted along with the study of ESPECIAL (Effects of Low Sodium Intake on the Antiproteinuric Efficacy of Olmesartan in Hypertensive Patients with Albuminuria; clinicaltrials.gov-No: NCT01552954), which enrolled 245 non-diabetic hypertensive patients using a randomization protocol [14]. All of the patients who participated in the present study provided written informed consent and agreed with the following protocol. First, 312 patients were screened from the outpatient clinics of 7 centers in Korea between March 2012 and March 2013. All patients fulfilled the following inclusion criteria: 19–75 years old; the use of antihypertensive medication or a diagnosis of hypertension; estimated glomerular filtration rate ≥ 30 mL/min/1.73 m^2^, which was calculated with the Modification of Diet in Renal Disease Study equation; random urine albumin-to-creatinine ratio ≥ 30 mg/g creatinine more than two times with a ≥ 1-week interval in the last 6 months; and an ability and willingness to provide written informed consent. The exclusion criteria were as follows: uncontrolled hypertension (blood pressure > 160/110 mmHg); pregnancy; serum potassium > 5.5 mEq/L; malignancy; diagnosis of cardiovascular disease within the last 6 months; contraindication to angiotensin II receptor blockers; diabetes mellitus; and continuous users of steroids or other immunosuppressive agents. This screening was conducted 8 weeks prior to study initiation.

After screening, all participants had to stop the use of blocking agents of the renin angiotensin system and diuretics, or were switched to antihypertensive agents of different categories until the initiation of the study. From the time of the study initiation, participants were prescribed olmesartan medoxomil (Daewoong Pharmaceutical Co. Ltd./Daiichi Sankyo Korea Co. Ltd., Seoul, Korea) at 40 mg, once per day for 16 weeks, at which time the study ended. Additionally, participants were randomly assigned to either conventional education or intensive education for a low-salt diet because the original aim of the study was to assess the proteinuria-lowering effects of intensive education in non-diabetic patients with olmesartan.

At the time of study initiation, clinical and laboratory parameters were obtained, including age, sex, weight, height, blood pressure, histories of dyslipidemia and cardiovascular disease (in adherence to the inclusion criteria), smoking, exercise, the use of medications including beta blockers, calcium channel blockers, statins, and antiplatelet agents, and several blood (hemoglobin, cholesterol, uric acid, and creatinine) and urine (sodium and albumin) findings. The body mass index was calculated as [weight (kg)/height (m^2^)]. Additionally, participants were asked to collect 24-h urine samples to assess albuminuria at 8 and 16 weeks.

Some of the additional urine samples (*n* = 229) were stored in a freezer at −70 °C for future measurement of urinary adiponectin, with informed consent from the participants. Total urinary adiponectin concentrations were measured in duplicates using a highly sensitive enzyme-linked immunosorbent assay (BioVendor, Brno, Czech Republic), according to the manufacturer’s protocol. Urinary adiponectin levels were adjusted for urinary creatinine concentration.

### Statistical analysis

All of the analyses and calculations were performed using STATA software (version 12.0, StataCorp LP, College Station, TX, USA). The data are presented as the mean ± standard deviations for continuous variables and as proportions for categorical variables. Based on variable distributions using histograms, the variables with non-normal distributions are expressed as the median (interquartile ranges). The chi-squared test was used to compare categorical variables. Comparisons between normally distributed continuous variables were performed using an analysis of variance or a post-hoc analysis of the least significant difference, according to the number of comparison groups. Comparisons between non-normally distributed continuous variables were performed by either the Kruskal-Wallis test or the Mann–Whitney *U* test, depending on the number of comparison groups. The relationship between adiponectin and albuminuria as continuous variables was assessed using Pearson’s correlation and linear regression analyses. Stepwise logistic regression models were tested to further examine the risks of macroalbuminuria (24-h albumin > 300 mg/day) or the disappearance of albuminuria (normoalbuminuria; 24-h albumin < 30 mg/day) according to the tertiles of adiponectin levels as follows: model 1, unadjusted for any covariate; model 2, adjusted for covariates with *P* < 0.1 in the univariate analysis; and model 3, adjusted for all of the covariates. To account for a possible nonlinear relationship with the outcomes, we also applied the fractional polynomials method and showed the relationship as a fitted curve [15]. A *P* value less than 0.05 was considered significant.

## Results

### Baseline characteristics

The baseline characteristics of the patients are shown and compared among the groups based on the urinary adiponectin level in Table 1. For the 229 subjects, the mean age was 50 years. All of the subjects were of Asian descent. The urinary adiponectin levels were different among the tertile groups with regard to several characteristics, including age, sex, smoking, exercise, statin use, hemoglobin, kidney function, and albuminuria. Intensive diet education was conducted less frequently in the second tertile group than in the other tertiles. All of the participants were examined for albuminuria at 8 and 16 weeks.

**Table 1:** Baseline characteristics of the study subjects

		Urinary adiponectin			
Variables	Total (*n* = 229)	1^st^ tertile (*n* = 76)	2^nd^ tertile (*n* = 77)	3^rd^ tertile (*n* = 76)	*P*
Age (years)	49.5 ± 13.33	46.1 ± 14.29	51.2 ± 11.51*	51.2 ± 13.56*	0.022
Male (%)	49.8	64.5	40.3**	44.7*	0.006
Body mass index (kg/m^2^)	25.4 ± 3.81	25.5 ± 4.05	25.3 ± 3.63	25.4 ± 3.78	0.956
Mean blood pressure (mmHg)	105.6 ± 9.35	104.7 ± 9.07	104.8 ± 8.94	107.3 ± 9.90	0.161
Dyslipidemia (%)	56.6	47.9	64.4*	57.5	0.132
History of cardiovascular disease (%)	3.5	2.6	5.2	2.7	0.614
Current smoking (%)	12.2	7.9	7.8	21.1*	0.016
Regular exercise (%)	52.4	56.6	59.7	40.8	0.043
Intensive diet education (%)	48.5	56.6	33.8**	55.3	0.007
Medication (%)					
Beta blocker	35.4	32.9	31.2	42.1	0.316
Calcium channel blocker	93.0	92.1	94.8	92.1	0.751
Statin	48.5	35.5	55.8*	53.9*	0.021
Antiplatelet agent	43.7	40.8	41.6	48.7	0.556
Blood findings					
Hemoglobin (g/dL)	14.0 ± 1.71	14.4 ± 1.60	14.0 ± 1.66	13.6 ± 1.77***	0.009
Cholesterol (mg/dL)	183.8 ± 35.27	181.8 ± 33.49	183.4 ± 35.09	186.3 ± 37.42	0.722
Uric acid (mg/dL)	6.4 ± 1.80	6.3 ± 1.80	6.1 ± 1.65	6.7 ± 1.93	0.192
Creatinine (mg/dL)	1.1 ± 0.41	1.0 ± 0.29	1.1 ± 0.39	1.3 ± 0.49***	<0.001
eGFR (ml/min/1.73 m^2^)	79.9 ± 24.62	88.9 ± 21.61	80.4 ± 21.89*	70.4 ± 26.75***	<0.001
Urine findings					
Adiponectin (μg/g creatinine)	34.5 (15.0–86.8)	11.2 (3.9–15.0)	34.5 (25.9–46.6)***	128.6 (86.5–202.9)***	<0.001
24-h sodium (mEq/day)	155.1 ± 68.94	162.1 ± 77.81	145.9 ± 55.61	157.5 ± 71.54	0.328
24-h albumin (mg/day)	550.0 (241.4–1286.1)	259.4 (134.3–517.2)	501.0 (268.0–1227.4)***	1317.0 (660.9–2419.5)***	<0.001

Comparisons were evaluated using the chi-squared test for categorical variables, the ANOVA test for normally distributed continuous variables (post hoc analysis of LSD between two groups), and the Kruskal-Wallis test for non-normally distributed continuous variables (Mann–Whitney *U* test between two groups). The 1^st^ tertile group served as a reference for comparison between two groups

^*^ *P* < 0.05; **P < 0.01; ***P < 0.001

eGFR, estimated glomerular filtration rate

### Correlation between urinary adiponectin and albuminuria

**Fig. 1:**
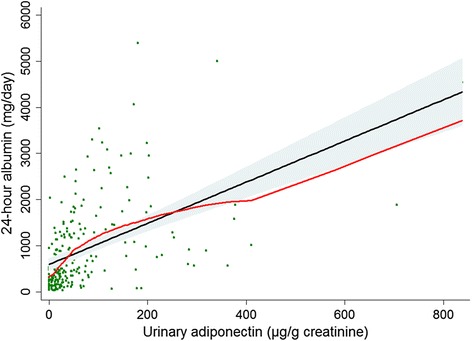
Scatter plot with the linear (*black*) and Lowess (*red*) regression curves between urinary adiponectin and the baseline 24-h albumin level

We first examined the relationship between urinary adiponectin and albuminuria at the time of study initiation (0 week). As shown in Fig. 1, the urinary adiponectin level was positively related to the albuminuria level (*r* = 0.529; *P* < 0.001). The Lowess line supported the linear relationship between urinary adiponectin and albuminuria. After adjusting for covariates, the adiponectin level was associated with the albuminuria level (*β* = 0.446; *P* < 0.001). To determine the odds ratio (OR) of macroalbuminuria at baseline, stepwise logistic regression models were used (Table 2). The risk of macroalbuminuria increased depending on the tertiles of urinary adiponectin, regardless of the effects of the covariates.

**Table 2:** Odds ratio of macroalbuminuria according to the tertiles of urinary adiponectin

	Model 1		Model 2		Model 3	
Adiponectin group	OR (95 % CI)	*P*	OR (95 % CI)	*P*	OR (95 % CI)	*P*
1^st^ tertile	1 (Reference)		1 (Reference)		1 (Reference)	
2^nd^ tertile	3.3 (1.68–6.47)	0.001	3.9 (1.68–9.02)	0.002	4.3 (1.72–10.54)	0.002
3^rd^ tertile	12.2 (4.95–29.93)	< 0.001	13.8 (4.77–40.21)	< 0.001	13.8 (4.34–43.62)	< 0.001

Model 1: unadjusted for any covariate

Model 2: adjusted for age, sex, dyslipidemia, smoking, exercise, diet education, statin, hemoglobin, and estimated glomerular filtration rate

Model 3: adjusted for all the covariates

OR, odds ratio; CI, confidence interval

### Risk of consistent macroalbuminuria according to adiponectin level

**Fig. 2:**
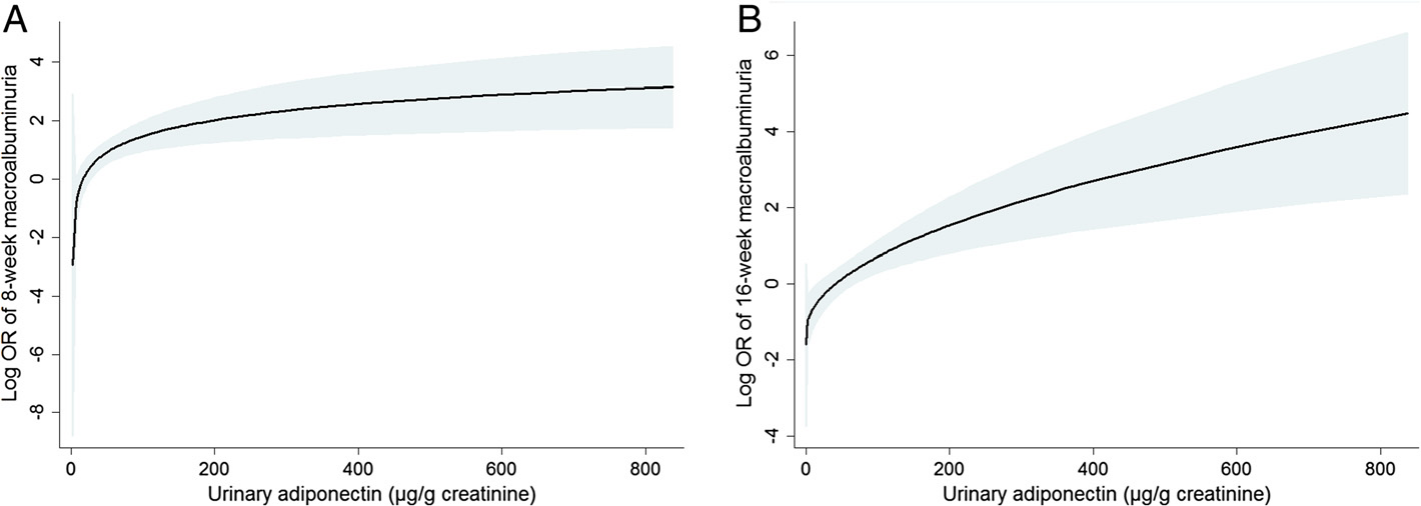
Fitted curve of the macroalbuminuria risk at 8 weeks (**a**) and 16 weeks (**b**), according to the urinary adiponectin level

At the time of the study initiation, a total of 159 subjects (69.4 %) had baseline macroalbuminuria. After conventional or intensive diet education, the proportion of macroalbuminuria decreased substantially. Among these subjects, 31.4 % and 43.4 % of them recovered from macroalbuminuria at 8 and 16 weeks, respectively. We categorized the trend of macroalbuminuria according to the tertiles of urinary adiponectin (Table 3). At 16 weeks, more than half of the subjects in the 1^st^ tertile group achieved a recovery from macroalbuminuria, whereas few subjects in the 3^rd^ tertile recovered. We initially explored the non-linear relationship between urinary adiponectin and the risk of consistent macroalbuminuria at 8 and 16 weeks (Fig. 2). The trend in this figure showed that the risk of consistent macroalbuminuria increased according to the increase in urinary adiponectin levels. Subsequently, the unadjusted and adjusted ORs of consistent 16-week macroalbuminuria were higher in the 3^rd^ tertile than in the 1^st^ tertile group (Table 4). The analysis of 8-week macroalbuminuria had a similar trend (see Additional file 1).

**Table 3:** Trends of albuminuria according to the urinary adiponectin levels in the participants with baseline macroalbuminuria

		8 weeks		16 weeks	
Goal	Adiponectin group	Achieved [*n* (%)]	Not achieved [*n* (%)]	Achieved [*n* (%)]	Not achieved [*n* (%)]
Albuminuria ≤ 300 mg/day	1^st^ tertile (n = 34)	19 (55.9)	15 (44.1)	22 (64.7)	12 (35.3)
	2^nd^ tertile (n = 56)	20 (35.7)	36 (64.3)	28 (50.0)	28 (50.0)
	3^rd^ tertile (n = 69)	11 (15.9)	58 (84.1)	19 (27.5)	50 (72.5)

**Table 4:** Implication of urinary adiponectin as a predictor of clinical outcome

		Model 1		Model 2		Model 3	
16-week outcome	Adiponectin group	OR (95 % CI)	*P*	OR (95 % CI)	*P*	OR (95 % CI)	*P*
Macroalbuminuria	1^st^ tertile	1 (Reference)		1 (Reference)		1 (Reference)	
	2^nd^ tertile	1.8 (0.76–4.41)	0.176	1.7 (0.65–4.68)	0.273	2.3 (0.80–6.86)	0.119
	3^rd^ tertile	4.8 (2.00–11.63)	<0.001	5.0 (1.73–14.48)	0.003	6.9 (2.06–22.98)	0.002
Normoalbuminuria	1^st^ tertile	16.9 (2.17–132.41)	0.007	16.5 (1.81–150.62)	0.013	13.0 (1.38–122.37)	0.025
	2^nd^ tertile	6.4 (0.76–54.74)	0.089	10.6 (1.05–107.46)	0.045	9.4 (0.89–99.42)	0.062
	3^rd^ tertile	1 (Reference)		1 (Reference)		1 (Reference)	

Model 1: unadjusted for any covariate

Model 2: adjusted for age, sex, dyslipidemia, smoking, exercise, diet education, statin, hemoglobin, and estimated glomerular filtration rate

Model 3: adjusted for all the covariates

OR, odds ratio; CI, confidence interval

**Fig. 3:**
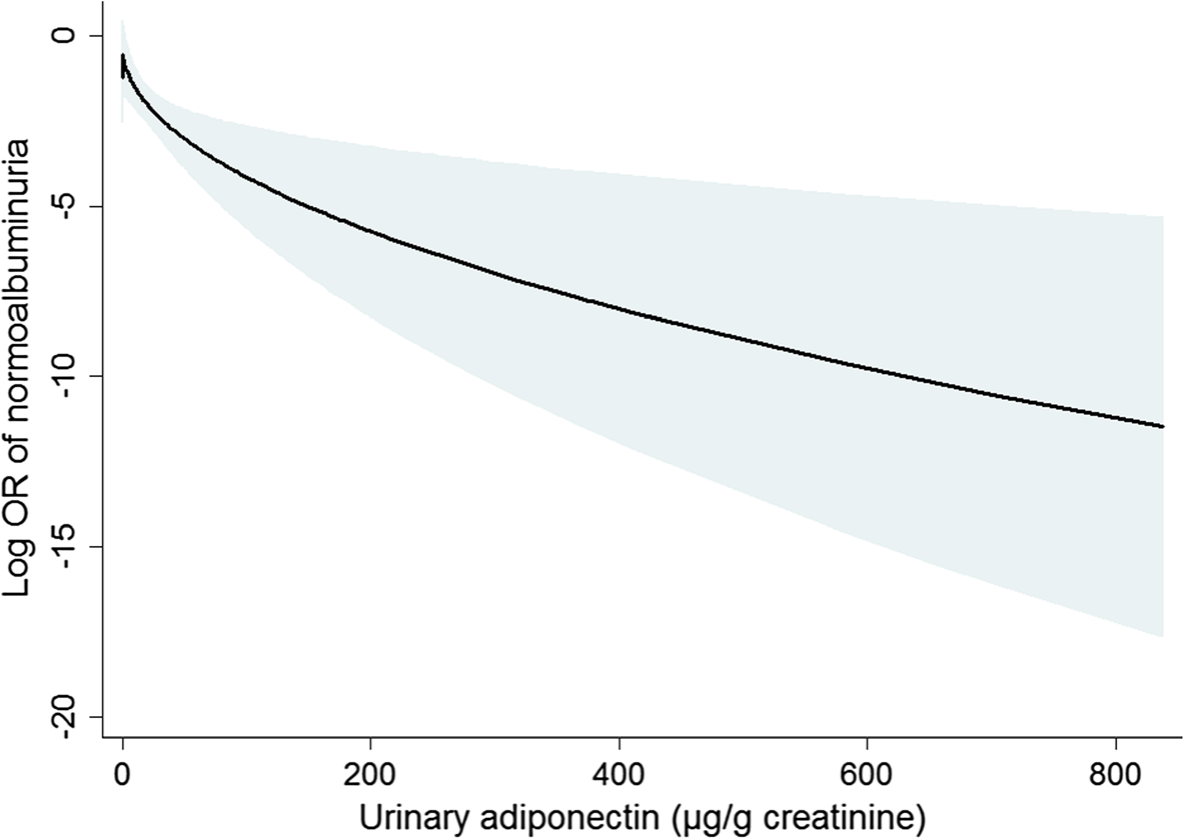
Fitted curve of the achievement of normoalbuminuria at 16 weeks, according to the urinary adiponectin level

Additionally, we examined the predictability of normoalbuminuria after treatment. At 8 and 16 weeks, albuminuria disappeared in 4.8 % and 9.2 % of participants, respectively. Figure 3 shows the inverse relationship between the urinary adiponectin level and the likelihood of subsequent normoalbuminuria. We calculated the unadjusted and adjusted ORs of the 16-week outcome after assigning a reference to the 3^rd^ tertile (Table 4). As a result, the subjects with low adiponectin levels achieved normoalbuminuria more frequently than did the subjects with high adiponectin levels. For the 8-week outcome, neither a non-linear relationship nor ORs could be calculated because of the low number of cases attaining normoalbuminuria at 8 weeks, and there were no such cases in the 3^rd^ tertile group.

## Discussion

Albuminuria has clinical implications for both cardiovascular and non-cardiovascular mortality. Previous studies have investigated whether adiponectin predicts albuminuria or its change. However, most of these studies used blood adiponectin, and the few studies that used urinary adiponectin included diabetic subjects alone or had a small sample size. The present study determined the relationship in a non-diabetic hypertensive cohort and supports the results observed in the diabetic patients included in other studies. Furthermore, urinary adiponectin was a strong predictor of treatment response (i.e., the reduction of albuminuria by diet education in the present trial). Based on these results, urinary adiponectin can be used as a biomarker of albuminuria or its trend in hypertensive patients without diabetes.

Several investigational studies have established the direct effect of adiponectin on the kidney. Ohashi et al. compared albuminuria between adiponectin-knockout and wild-type mice after subtotal nephrectomy [16] found that albuminuria was increased to a greater extent in the knockout model compared with the wild-type model. Additionally, the authors found that the markers, such as oxidative stress, inflammation, and fibrosis, were different between the models and suggested that adiponectin contributes to kidney injury via these factors. Using adiponectin-knockout mice, Sharma et al. further focused on the adiponectin-stimulated 5’ adenosine monophosphate-activated protein kinase (AMPK) of podocytes, and the subsequent stabilization of glomerular permeability and the reduction of oxidant stress [7]. These subsequent changes were identified with zonula occludens-1 and nicotinamide adenine dinucleotide phosphate (NADPH) oxidase, respectively. The beneficial role of adiponectin was also confirmed in other *in vivo* models [17, 18] and in other kidney tissues, such as the tubule and vessel [12, 17, 19].

When the direct effect of adiponectin is considered, it is possible to observe a strong relationship between adiponectin and albuminuria in human studies. However, the relationship with blood adiponectin is complicated and can vary according to kidney state or diabetic type [8–10]. Although the reason for the complicated relationship has not been fully explored, this feature can affect the other associations, such as cardiovascular morbidity and mortality. As a result, some studies have found no correlation between blood adiponectin and cardiovascular markers [20]. In contrast, one study revealed an inverse U-shaped relationship between blood adiponectin and all-cause mortality in patients with end-stage renal disease [21]. In addition to the non-invasive nature of urine sampling, urinary adiponectin has an advantage over blood adiponectin because it is positively and consistently related to the albuminuria level [3, 11–13]. However, it is too early to consider urinary adiponectin a successful biomarker of albuminuria because there are few studies on this issue, particularly studies including non-diabetic subjects. We believe the present results meet a need related to this issue.

We did not separate the molecular isoforms of adiponectin. Some reports have suggested that high molecular weight adiponectin is more reliable than the low molecular weight isoform in predicting outcomes [22]. However, these reports have mainly concerned blood adiponectin, not the urinary counterpart. Adiponectin exists largely as the low molecular weight isoform in urine [12], and there are reports that only low molecular weight adiponectin, and not the high molecular weight isoform, has anti-inflammatory property [23].

Increased glomerular permeability due to podocyte injury can induce the hyper-filtration of adiponectin into urine. Monomeric and trimeric adiponectin (i.e., low molecular weight) have a molecular weight that is smaller than or similar to that of albumin. Accordingly, urinary adiponectin is likely to precede the onset of albuminuria. The present study shows that urinary adiponectin can predict the response of albuminuria to a certain treatment. A few studies have also found that the adiponectin level was altered by treatment (i.e., angiotensin II receptor blockers) and its response, although those studies used blood adiponectin [24]. In view of the results achieved so far, both blood and urinary adiponectin levels can be useful independent biomarkers for the treatment response of albuminuria. As previously stated, urinary adiponectin is more accessible than blood adiponectin because of the intrinsic nature of the test and the consistent results that have been observed across several diseases.

Although the present results are informative, this study has some limitations. First, the study design, which involves observing correlations, limits the drawing of conclusions based on causality. However, the main aim of the present study was to determine whether urinary adiponectin is a successful biomarker; thus, the current design does not significantly hamper this aim. Second, the overall duration of treatment response (i.e., 8 and 16 weeks) was relatively short. However, 58 % of the total participants (43 % of participants with baseline macroalbuminuria) achieved a reduction of macroalbuminuria at 16 weeks. This result indicates that the treatment duration was sufficient to obtain results for analysis. Third, we did not consider other valuable parameters, such as patient compliance, weight change, and the control of blood pressure in the following course of study; these parameters might also affect proteinuria and its response.

## Conclusions

Urinary adiponectin was a strong predictor of albuminuria, as previously found in other clinical settings. Furthermore, it was correlated with the real response of albuminuria after albuminuria reduction was implemented. Based on the results presented by our and other studies, urinary adiponectin can be used as a biomarker for albuminuria. Because albuminuria alone is not an accurate prognostic indicator of several outcomes, additional studies are needed to address whether urinary adiponectin has predictive power in these outcomes.

## Additional file

Additional file 1:
**Urinary adiponectin in predicting 8-week macroalbuminuria.** (PDF 91 kb)

## Abbreviations

OR: Odds ratio
AMPK: 5’ adenosine monophosphate-activated protein kinase
NADPH: Nicotinamide adenine dinucleotide phosphate
