# Supplementary material for: Urinary adiponectin and albuminuria in non-diabetic hypertensive patients: an analysis of the ESPECIAL trial
Source: BMC Nephrol. 2015 Aug 1;16:123. doi: 10.1186/s12882-015-0124-3 (PMC4522110; doi:10.1186/s12882-015-0124-3)
Supplement: Additional file 1: — Urinary adiponectin in predicting 8-week macroalbuminuria. (PDF 91 kb) [file 12882_2015_124_MOESM1_ESM.pdf]

Additional file 1. Urinary adiponectin in predicting 8-week macroalbuminuria

| | | Model 1 | | Model 2 | | Model 3 | |
|---|---|---|---|---|---|---|---|
| 8-week outcome | Adiponectin group | OR (95% CI) | *P* | OR (95% CI) | *P* | OR (95% CI) | *P* |
| Macroalbuminuria | 1st tertile | 1 (Reference) | | 1 (Reference) | | 1 (Reference) | |
| | 2nd tertile | 2.3 (0.96–5.44) | 0.063 | 2.3 (0.83–6.29) | 0.109 | 2.8 (0.91–8.73) | 0.072 |
| | 3rd tertile | 6.7 (2.62–17.01) | < 0.001 | 6.0 (1.91–18.57) | 0.002 | 11.3 (2.92–43.95) | < 0.001 |

Model 1: unadjusted for any covariate.

Model 2: adjusted for age, sex, dyslipidemia, smoking, exercise, diet education, statin, hemoglobin, and estimated glomerular filtration rate.

Model 3: adjusted for all the covariates.

OR, odds ratio; CI, confidence interval.
